# Can ^68^Ga-prostate specific membrane antigen positron emission tomography/computerized tomography provide an accurate lymph node staging for patients with medium/high risk prostate cancer? A diagnostic meta-analysis

**DOI:** 10.1186/s13014-020-01675-4

**Published:** 2020-10-01

**Authors:** Lei Peng, Jinze Li, Chunyang Meng, Jinming Li, Chengyu You, Dandan Tang, Tangqiang Wei, Wei Xiong, Yunxiang Li

**Affiliations:** 1 Department of Urology, The Second Affiliated Hospital of Medical College, Nanchong Central Hospital, North Sichuan Medical College, Nanchong, 63700 Sichuan China; 2 Department of Urology, The Affiliated Hospital of Medical College, Nanchong Central Hospital, North Sichuan Medical College, Nanchong, 63700 Sichuan China; 3 Department of Cardiothoracic Surgery, Shenzhen People’s Hospital, Affiliated Hospital of Jinan University, Shenzhen, 518000 Guangdong China

**Keywords:** Prostate cancer, Lymph node, ^68^Gallium- prostate-specific membrane antigen, Meta-analysis

## Abstract

**Objective:**

This article aims to evaluate the diagnostic value of ^68^Gallium-PSMA positron emission tomography/computerized tomography (^68^Ga-PSMA PET/CT) for lymph node (LN) staging in patients with prostate cancer (PCa) by a meta-analysis of diagnostic tests.

**Methods:**

We systematically retrieved articles from Web of Science, EMBASE, Cochrane Database, PubMed. The time limit is from the creation of the database until June 2019, and Stata 15 was used for calculation and statistical analyses.

**Results:**

Sensitivity, specificity, positive and negative likelihood ratio (PLR, NLR), diagnostic odds ratio (DOR) and 95% confidence intervals (CI) be used to evaluate the diagnostic value. A total of 10 studies were included in our meta-analysis, which included 701 individuals. The results of each consolidated summary are as follows: sensitivity of 0.84 (95% CI 0.55–0.95), specificity of 0.95 (95% CI 0.87–0.98), PLR and NLR was 17.19 (95% CI 6.27, 47.17) and 0.17 (95% CI 0.05–0.56), respectively. DOR of 100 (95% CI 18–545), AUC of 0.97 (95% CI 0.95–0.98).

**Conclusion:**

Our study demonstrates that ^68^Ga-PSMA PET/CT has a high overall diagnostic value for LN staging in patients with moderate and high-risk PCa. But our conclusions still require a larger sample size, multi-center prospective randomized controlled trial to verify.

## Introduction

Prostate cancer (PCa) is the second most common cancer and the fifth leading cause of cancer death in developed countries [1]. Although the clinical symptoms of early PCa are not obvious, pelvic lymph node (LN) and bone metastasis are also prone to occur [2]. About 15% of patients with medium and high-risk prostate cancer were found to have harbor lymph node invasion when performing pelvic lymph node dissection (PLND) during radical prostatectomy (RP) [3]. PLND is undoubtedly the most reliable way to determine whether there is LNI in PCa, but this method is both invasive and expensive [4]. And complications such as lymphocele, lymphedema are also headaches [5]. Before performing PLND, it is necessary to routinely use nomograms to predict the extent of lymph node invasion before surgery. According to these nomograms, patients with a serum prostate-specific antigen (PSA) of over 10 ng/mL, a Gleason score of over 6, or a stage T3 tumour (according to the Tumour, Nodes, and Metastases [TNM] staging system) defined by digital rectal examination (DRE), have a 5–65% risk of lymph node involvement [6–8]. According to the EAU guidelines, patients with moderate to high risk PCa who have an LN-positive estimated risk of more than 5% should undergo extend pelvic lymph node dissection (ePLND) [9]. Therefore, accurate assessment of LN staging before surgery is important for the operation and patient’s prognosis.

Presurgical computerized tomography (CT) scan or magnetic resonance imaging (MRI) is helpful for LN staging, but with limited accuracy [10]. Prostate-specific membrane antigen (PSMA) is a surface protein that is expressed in almost normal prostate tissues, and its expression level is higher in PCa tissues [11]. In recent years, PSMA has been introduced into positron emission tomography (PET) imaging [12], ^68^Gallium-PSMA positron emission tomography/computerized tomography (^68^Ga-PSMA PET/CT) has been proven to be better than ordinary imaging tests in detecting metastatic PCa [13]. Related studies have also shown that ^68^Ga-PSMA PET/CT is more sensitive for recurrent PCa with a low prostate-specific antigen (PSA) [14, 15].

Till now, some scholars have done some research on the accuracy of ^68^Ga-PSMA PET/CT to determine the lymph node staging of PCa preoperative, but it’s still unclear how its diagnostic value is. The diagnostic accuracy of ^68^Ga-PSMA PET/CT to determine lymph node staging of PCa preoperative remains unclear based on the current literature. The current study aims to establish the status of ^68^Ga-PSMA PET/CT in PCa's LN staging.

## Methods

### Literature search and eligibility criteria

A systematic search of the published literature using Web of Science, EMBASE, Cochrane Database, PubMed was performed. We have selected relevant literature from the creation of the database to June 2019. And used prostate cancer, Gallium, lymph node, positron emission tomography/computerized tomography, prostate-specific membrane antigen as the search terms and the search language was limited to English. We also searched for the relevant bibliography to avoid omissions.

The studies that were included in our research should meet the demands as follows: patients diagnosed with LN metastatic PCa using the gold standard pathological biopsy, studies with the diagnostic value of ^68^Ga-PSMA PET/CT reflected in the studies, and studies with sufficient data on true positive (TP), false positive (FP), false negative (FN), and true negative (TN). For duplicate articles, low-quality research, letters, reviews, case report, we analyze and exclude respectively. This process was assessed by two authors (PL and LJZ), independently.

### Data extraction

We incorporate the following data from each article into the meta-analysis: author, publication year, study design, patient recruitment time, lymph node dissection, average patient age, preoperative PSA, patient population characteristics: high/medium risk. The PCa and health group was diagnosed as positive and negative by ^68^Ga-PSMA PET/CT to calculate the number of true positives, false positives, true negatives, and false negatives. Data extraction was performed by two authors (PL and LJZ) with any discrepancies resolved by a third author (WTQ).

### Quality evaluation

The Quality Assessment of Diagnostic Accuracy Studies 2 (QUADAS-2) tool was used to evaluate the quality of included studies [13]. Key domains are assessed to determine the risk of bias and applicability. Signalling questions are included to facilitate judgements, with the risk being low if all signaling answer for a domain is 'yes', and if the answer to any question is 'no' suggesting potential bias exists. Concerns about applicability are determined as 'low', 'high', or 'unclear'.

### Statistical analysis

We used Stata 15 (StataCorp LP, University City, Texas, USA) for statistical analysis. Q test and chi-square tests were used to verify the heterogeneity between the included works of literature. If I^2^ > 50%, the differences between the literature were considered significant [16]. We used a bivariate model to calculate the pooled sensitivity, specificity, positive and negative likelihood ratios (PLRs and NLRs), diagnostic odds ratio (DOR) and the 95% confidence interval (CI) [17]. We calculated the area under the receiver operator characteristic curve (SROC, AUC). AUC varied from 0.5 to 1. If the area was equal to 1, then a diagnostic test was extremely valuable. If the area was 0.5, then the diagnostic ability was considered as poor [18]. Deeks’s funnel plot was used to assess the publication bias, and Fagan plots showed the relationship between the prior probability, the likelihood ratio, and the posterior test probability [19]. *P* < 0.05 was considered statistically significant.

## Results

### Study selection and study characteristics

**Fig. 1:**
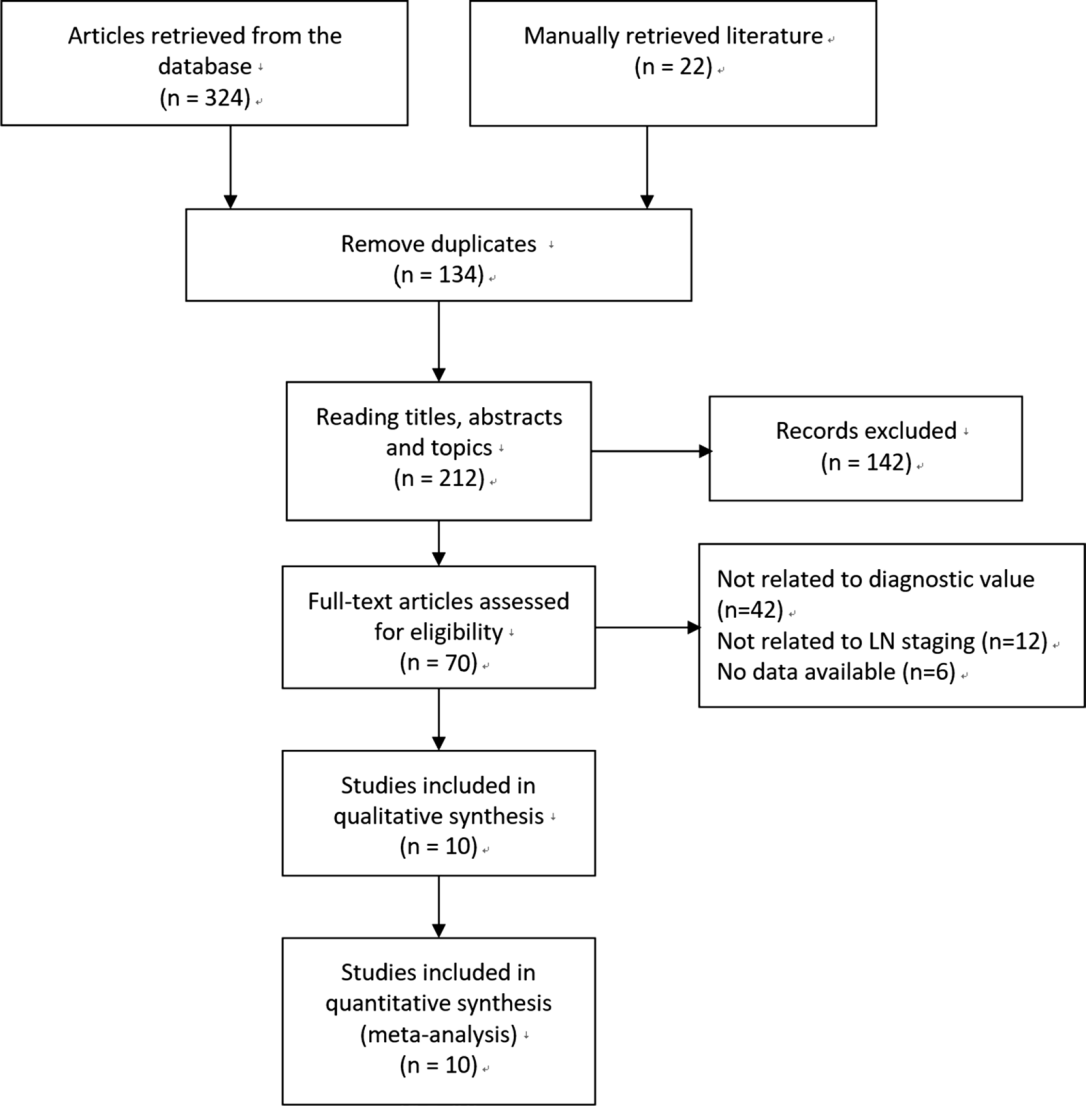
Flow diagram of studies selection process

The literature search selection steps retrieval in Fig. 1. Initially, we retrieved a total of 324 articles in the selected database and manually retrieved 22 articles by referring to relevant literature citations. In these studies, we excluded 134 duplicates records. By analyzing titles, abstracts, and topics, 142 articles were also excluded. Through full-text analysis and assessment of eligibility, 42 articles that were not related to diagnostic value, 12 articles that were no related to LN staging, and 6 studies that had no access data. Finally, we included 10 studies in our meta-analysis [2, 12, 13, 20–26].

In Table 1, we presented the basic data of the articles included in the meta-analysis of ^68^Ga-PSMA PET/CT for LN staging of PCa preoperative. These articles were published between 2016 and 2019. A total of 701 sample individuals were included in our analysis. Most of them are single-center retrospective studies in regions including Asia (China, India, and Turkey), Europe (Germany) and Australia. At the same time, we also display the age, PSA, patient characteristics and other information of patients included in each study.

**Table 1:** Characteristics of the included studies in the meta-analysis

References	Country	Study design	Study population	Recruiting time	Uptake time (min)	Lymphade-nectomy	Age Median (range) Median (IQR^e^)	PSA^d^ median (range) Median (IQR)	Patient characteristics
Budaus [25]	Germany	Retrospective	Single center	June. 2014 to Mar. 2015	NA	ePLND^f^	63 (44–75)	8.8 (1.4–376)	High risk
Herlemann [20]	Germany	Retrospective	Single center	Jan. 2014 to Aug. 2015	60	PLND^g^	70.5 (59–80)^b^	56 (3.3–36.3)^b^	4 intermediate risk and 16 high risk
Maurer [24]	Germany	Retrospective	Single center	Dec. 2012 to Nov. 2014	59.8 ± 17.8^a^	PLND	66 (45–84)	11.6 (0.57–244)	42 intermediate risk and 88 high risk
Gupta [22]	India	Retrospective	Single center	Dec. 2014 to Dec. 2015	Nearly 60	ePLND	NA	NA	High risk
Obek [21]	Turkey	Retrospective	Single center	July. 2014 to Oct. 2015	45–60	ePLND	64 ± 6.0^a^	26.5 ± 21.4^a^	44 high risk and 7 very high risk
Van Leeuwen [26]	Australia	Prospective	Single center	Apr. to Oct. 2015	60	ePLND	65 (60–71)	65 (55–82)	3 intermediate risk and 27 high risk
Zhang [12]	China	Retrospective	Single center	Mar. to July. 2017	60	PLND	69 (55–82)	37.25 (7.2–348)	17 intermediate risk and 25 high risk
Van Leeuwen [2]	Australia	Retrospective	Single center	Feb. 2015 to Oct. 2017	60	ePLND	NA	9.4	140 intermediate risk and high risk
Yilmaz [23]	Turkey	Retrospective	Single center	May. 2016 to Apr. 2018	Approximately 60	rLND^c^	62.8 (49–73)^b^	12 (2.4–32)^b^	15 intermediate risk and 6 high risk
Yaxley [13]	Australia	Retrospective	Single center	July. 2014 to Sep. 2017	45–60	PLND	68 (44–80)	7.6 (1.5–51)	85 intermediate risk and 123 high risk

^a^Mean ± SD

^b^Mean (range)

^c^rLND, regional lymph node dissection

^d^PSA, prostate specific antigen

^e^IQR, interquartile range

^f^ePLND, extend pelvic lymph node dissection

^g^PLND, pelvic lymph node dissection

### Quality assessment

Based on the evaluation results of the QUADAS-2 scale, we reflected the quality evaluation results of each article in Table 2. The final score of each article was 11 or better, and 7 of them received quite high scores.

**Table 2:** Summary estimated of diagnostic performance of the ^68^Ga-PSMA PET/CT for lymph node staging in patient with Prostate Cancer

References	Sample size	TP^a^	FP^b^	FN^c^	TN^d^	Quality
Budaus [25]	30	33	0	67	100	11
Herlemann [20]	34	91	33	9	67	10
Maurer [24]	130	66	1	34	99	11
Gupta [22]	12	100	20	0	80	10
Obek [21]	51	53	14	47	86	10
Van Leeuwen [26]	30	64	5	62	94	11
Zhang [12]	42	93	4	7	96	9
Van Leeuwen [2]	140	60	12	40	88	10
Yilmaz [23]	24	38	6	62	94	10
Yaxley [13]	208	38	6	62	94	11
Pooled analysis	Inconsistency (I^2^) (95% CI)		99% (98–99)			
	Sample Size		701			
	SEN^e^ (95% CI)		0.84 (0.55, 0.95)			
	SPE^f^ (95% CI)		0.95 (0.87, 0.98)			
	PLR^g^ (95% CI)		17.2 (6.3, 47.2)			
	NLR^h^ (95% CI)		0.17 (0.05, 0.56)			
	DOR^i^ (95% CI)		100 (18, 545)			
	Youden Index		0.79			

^68^Ga-PSMA PET/CT, ^68^Gallium-PSMA positron emission tomography/computerized tomography

^a^TP, true positive

^b^FP, false positive

^c^FN, false negative

^d^TN, true positive

^e^SEN, Sensitivity

^f^SPE, Specificity

^g^PLR, Positive Likelihood Ratio

^h^NLR, Negative Likelihood Ratio

^i^DOR, Diagnostic Odds Ratio

### Pooled diagnostic values

**Fig. 2:**
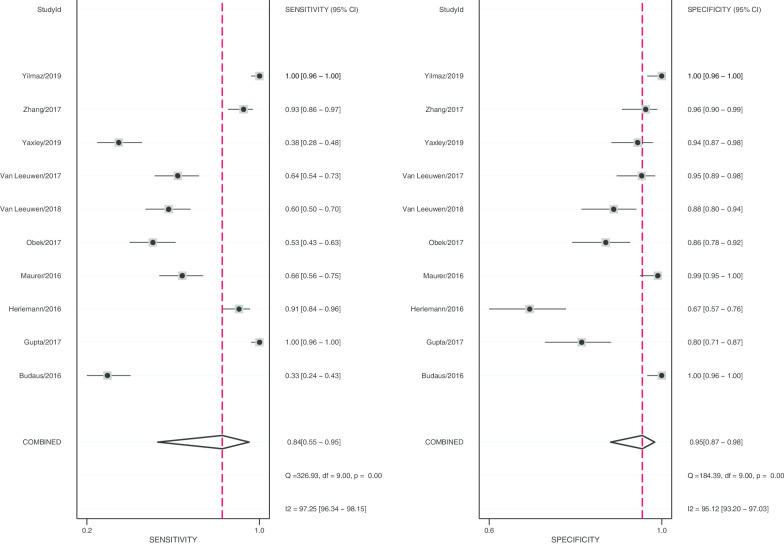
Forest plot of pooled sensitivity and specificity of ^68^Ga-PSMA PET/CT for lymph node staging in patient with prostate cancer

**Fig. 3:**
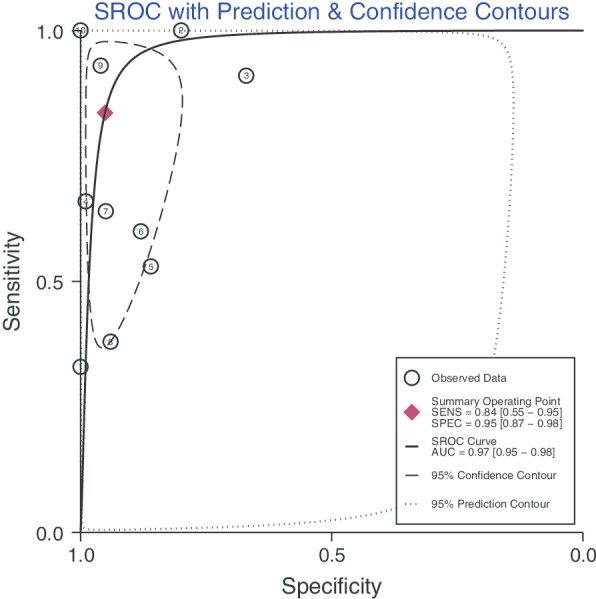
The SORC curve of ^68^Ga-PSMA PET/CT for lymph node staging in patient with prostate cancer

**Fig. 4:**
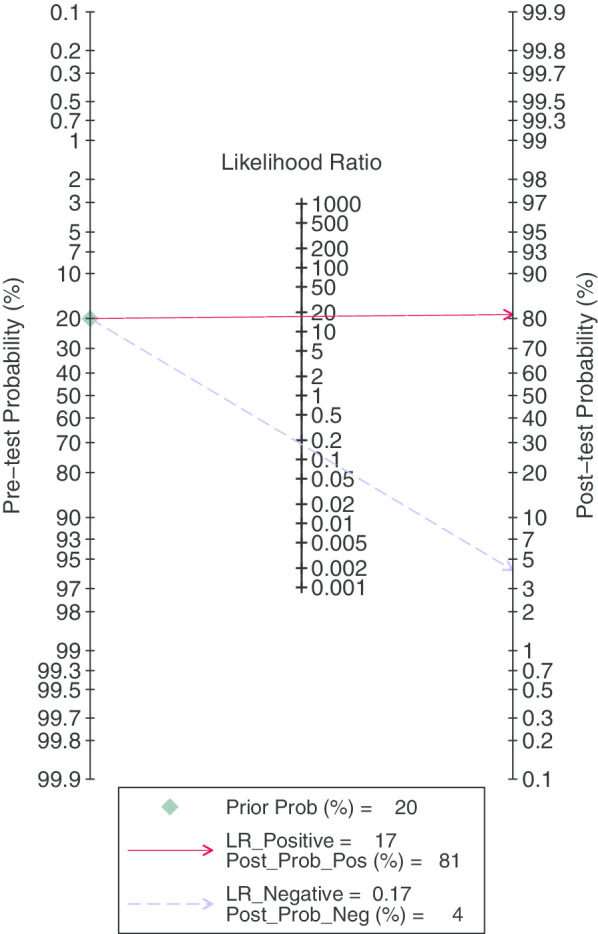
Fagan diagram evaluating the overall diagnostic value of ^68^Ga-PSMA PET/CT for lymph node staging in patient with prostate cancer

After statistical tests, the value of I^2^ is greater than 50%, it can be considered that the included documents have high heterogeneity. Therefore, a random effect model is used to combine sensitivity and specificity, thereby obtaining more conservative results. The diagnostic value is shown in Table 2. The pooled sensitivity and specificity were recorded as 0.84 (95% CI 0.55–0.95) and 0.95 (95% CI 0.87–0.98, Fig. 2), respectively. The Youden index was 0.79. The pooled PLR and NLR was 17.19 (95% CI 6.27–47.17) and 0.17 (95% CI 0.05–0.56), and DOR of 100 (95% CI 18–545). The overall SORC curve is presented in Fig. 3, with an AUC of 0.97 (95% CI 0.95–0.98). Figure 4 is shown in the Fagan plot. The prior probability was 20%, and the post-test probability was 81% for LR-positive and 4% for LR-negative. The diagnostic accuracy for detecting ^68^Ga-PSMA PET/CT for LN staging in PCa was found to be generally better.

### Publication bias

**Fig. 5:**
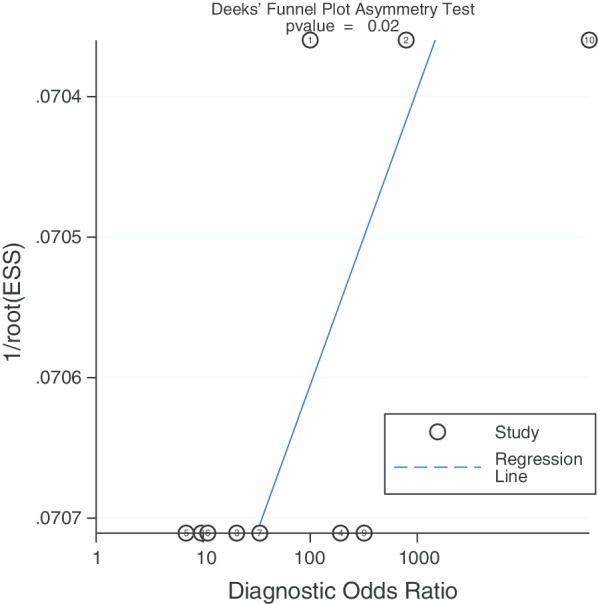
Deek’s funnel plot to evaluate the publication bias

The publication bias (*P* = 0.02, Fig. 5) was shown in Deek’s funnel plot.

### Heterogeneity and sensitivity analysis

According to the results of the forest plot, the heterogeneity of ^68^Ga-PSMA PET/CT was high in both sensitivity (I^2^ = 97.25%) and specificity (I^2^ = 95.12%). Due to the small number of studies we included (n = 10) that prevented meta-regression from being implemented, a random-effects model was used to pool the data of ^68^Ga-PSMA PET/CT.

### Meta regression and subgroup analysis

**Fig. 6:**
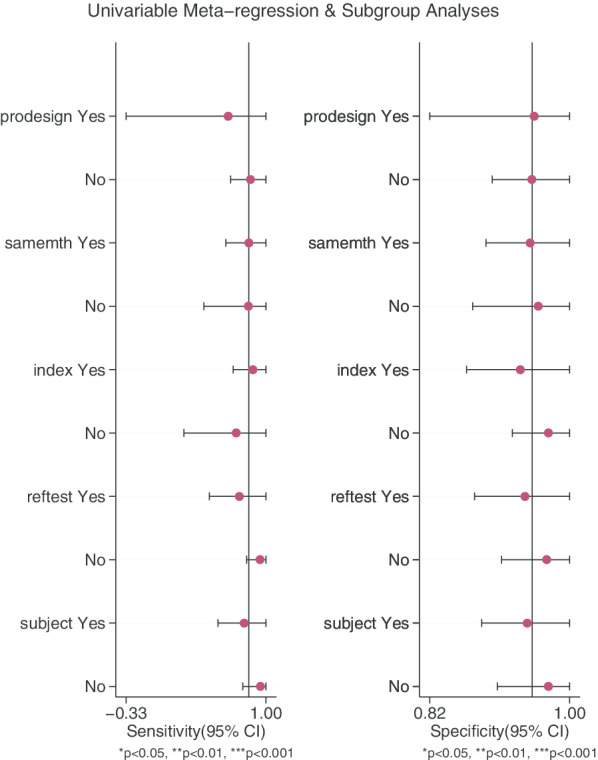
Meta regression and subgroup analysis

Meta-regression and subgroup analysis were performed on study design (predesign), gold standard selection and description (samemth and reftest), diagnostic test to be evaluated (index), and patient characteristics (subject). It can be seen from the forest plot that in the study we included, the five independent variables had no statistically significant effects on sensitivity and specificity (P > 0.05, Fig. 6).

## Discussion

Based on the currently published research, our meta-analysis fills the gap of ^68^Ga-PSMA PET/CT in the diagnosis of middle/high-risk prostate cancer lymph node staging to a certain extent. We found that the indicators and results of ^68^Ga-PSMA PET/CT show a satisfactory diagnostic efficacy.

In previous treatments, LN staging of prostate cancer patients can only be determined by performing PLND according to the situation during the operation. However, this method is not always feasible [27]. Clinically, the use of PLND is not always feasible, being restricted for the following reasons: not every patient will receive PLND, limited anatomical locations performed, and consideration regarding complications [28–30], resulting in suboptimal lymph node staging. ^68^Ga-PSMA PET/CT is a more accurate method to determine the LN stage of PCa patients before surgery [31], which can be incorporated into clinical practice. The common lymph node metastasis sites for prostate cancer are obturator lymph node, internal iliac lymph node, external iliac lymph node, common iliac lymph node, presacral lymph node, abdominal aortic lymph node and mediastinum, supraclavicular lymph node. Studies have shown that the most common lymph node metastasis sites for prostate cancer are external iliac lymph nodes and obturator lymph nodes, with the median percentages being 12.2% and 11.6%, respectively [32]. Based on the current evidence, the diagnostic performance of auxiliary imaging and nomograms tool for prostate cancer lymph node metastasis still has limitations, especially for lymph nodes < 5 mm. Preoperative assessment of the degree of lymph node metastasis can guide the selection of surgical procedures, which can further assist the management of patients after radical prostatectomy [33–35]. Therefore, it is particularly important to find a non-invasive and better diagnostic method.

Our meta-analysis included 10 studies from different countries and regions, involving a total of 701 patients. The pooled specificity of 0.95 (95% CI 0.87–0.98) for ^68^Ga-PSMA PET/CT. But pooled sensitivity was recorded as 0.84 (95% CI 0.55–0.95), which is not enough to convince us to give up the remaining 20% of the patient. The Youden index was 0.79. AUC was 0.97 (95% CI 0.95–0.98), which was in line with our initial predictions. Through these comprehensive indicators, we can think that ^68^Ga-PSMA PET/CT has better diagnostic efficacy in preoperative LN staging in patients with prostate cancer. In the past few decades, CT and MRI have been used to determine the LN staging before radical prostatectomy, but their accuracy rate is still low compared to the gold standard [9]. Hovels et al. Performed a meta-analysis of 24 studies to assess CT/MRI for preoperative evaluation of LN staging. In his research, for CT, pooled sensitivity and specificity was 0.42 (95% CI 0.26–0.56) and 0.82 (95% CI 0.8–0.83) respectively. For MRI, pooled sensitivity and specificity was 0.39 (95% CI 0.22–0.56) and 0.82 (95% CI 0.79–0.83) [36]. From this point of view, the performance of CT and MRI in judging LN staging is not satisfactory. If clinicians rely on CT or MRI, they will easily make the wrong decision on the patient’s condition. In our analysis, we could see that two studies have lower sensitivity, 0.38(95%: 0.28–0.48) and 0.33 (95% CI 0.24–0.43) respectively [13, 25]. The reason for this analysis was that, due to the technical level of the test, the sample size, and bias between the samples, it might have lead to different final results. The specificity value provided in one study is significantly lower [20]. We thought that the main reason is that the patients included in Herlemann’s [20] study received different PLNDs. Among them, 20 received primary PLND and 14 received secondary PLND, which may be the main reason for the lower specificity. Multi-parameter magnetic resonance (mpMR) also plays a large role in the preoperative evaluation of prostate cancer, especially in judging extraprostatic extension (EPE) of the tumor, invasion of the seminal vesicle (SVI) [23]. In a retrospective study by Van Leeuwen et al. They also compared the diagnostic accuracy of mpMR and ^68^Ga-PSMA PET/CT for LN metastasis in a patient with intermediate high-risk PCa. The sensitivity was 14% and 53%, respectively, and the specificity was 99% and 88%, respectively [2]. All of these indicated that ^68^Ga-PSMA PET/CT has better diagnostic efficacy and was expected to be popularized and used in clinical.

The higher the value of DOR, the better the diagnostic value of this diagnostic method. In our study, The DOR value was 100 (95% CI 18–545), indicating that the overall accuracy was high. Pooled PLR and NLR value was 17.19 (95% CI 6.27–47.17) and 0.17 (95% CI 0.05–0.56), respectively. This can be understood as the probability of ^68^Ga-PSMA PET/CT correctly judging LN metastasis is 17 times that of misjudging, and the probability of correctly judging LN non-metastasis is 0.17 times that of misjudging. At the same time, we also noticed that the publication bias shown by Deek’s funnel plot (Fig. 5) has a P-value of 0.02. It is understandable that most of the articles we included were retrospective trials, which led to the results. In addition, meta regression and subgroup analysis showed that there is no statistical difference between the above-mentioned influencing factors for the obtained sensitivity and specificity values, excluding the influence of other factors on the results. From the data results, it is worth trying to use ^68^Ga-PSMA PET/CT to diagnose LN staging in patients with PCa.

We perform this meta-analysis strictly according to PRISMA checklist [37]. However, there are still some limitations in our meta-analysis. First, most of the studies we included were single-center retrospective studies, and the existence of selection bias may affect our judgment. Second, the sample populations included in these articles are only Asia, Europe, and Australia, so population bias is unavoidable. Third, the sample size is too small. The current research is not registered, and there may be small deviations, but we still strictly follow the steps of systematic reviews. Due to the clinical application of ^68^Ga-PSMA PET/CT in the future, there is not a sufficiently large sample size to be included in our meta-analysis. Compared with PLND, although ^68^Ga-PSMA PET/CT has only moderate sensitivity and better specificity, it can perform relatively accurate LN staging of detected PCa patients. At this point, ^68^Ga-PSMA PET/CT is due to any other imaging examinations, but due to many limitations, our conclusions still require a larger sample size, multi-center prospective randomized controlled trial to verify.

## Conclusion

Based on the current evidence, ^68^Ga-PSMA PET/CT is bound to have a strong performance in the diagnosis of medium/high-risk prostate cancer lymph node staging due to its relatively excellent diagnostic accuracy. However, in a short period of time in the future, limited by the capacity of different medical institutions and the development of regional economic levels, it will take some time to fully demonstrate its advantages in the field of prostate cancer. Further studies in ^68^Ga-PSMA PET/CT are needed, and efforts are warranted to improve understanding and intervention of these diagnostic deviations and effectiveness.

## Data Availability

All data generated and analysed during this study are included in this published article.

## References

[1] Bray F, Ferlay J, Soerjomataram I, Siegel RL, Torre LA, Jemal A (2018). Global cancer statistics 2018: GLOBOCAN estimates of incidence and mortality worldwide for 36 cancers in 185 countries. CA Cancer J Clin.

[2] van Leeuwen PJ, Donswijk M, Nandurkar R, Stricker P, Ho B, Heijmink S, Wit EMK, Tillier C, van Muilenkom E, Nguyen Q (2019). Gallium-68-prostate-specific membrane antigen ((68) Ga-PSMA) positron emission tomography (PET)/computed tomography (CT) predicts complete biochemical response from radical prostatectomy and lymph node dissection in intermediate- and high-risk prostate cancer. BJU Int.

[3] Gandaglia G, Fossati N, Zaffuto E, Bandini M, Dell'Oglio P, Bravi CA, Fallara G, Pellegrino F, Nocera L, Karakiewicz PI (2017). Development and internal validation of a novel model to identify the candidates for extended pelvic lymph node dissection in prostate cancer. Eur Urol.

[4] Hovels AM, Heesakkers RA, Adang EM, Jager GJ, Strum S, Hoogeveen YL, Severens JL, Barentsz JO (2008). The diagnostic accuracy of CT and MRI in the staging of pelvic lymph nodes in patients with prostate cancer: a meta-analysis. Clin Radiol.

[5] Fossati N, Willemse PM, Van den Broeck T, van den Bergh RCN, Yuan CY, Briers E, Bellmunt J, Bolla M, Cornford P, De Santis M (2017). The benefits and harms of different extents of lymph node dissection during radical prostatectomy for prostate cancer: a systematic review. Eur Urol.

[6] Harbin AC, Eun DD (2015). The role of extended pelvic lymphadenectomy with radical prostatectomy for high-risk prostate cancer. Urol Oncol.

[7] Heesakkers RAM, Hövels AM, Jager GJ, van den Bosch HCM, Witjes JA, Raat HPJ, Severens JL, Adang EMM, van der Kaa CH, Fütterer JJ (2008). MRI with a lymph-node-specific contrast agent as an alternative to CT scan and lymph-node dissection in patients with prostate cancer: a prospective multicohort study. Lancet Oncol.

[8] Silberstein JL, Laudone VP. Pelvic Lymph Node Dissection for Prostate Cancer. In: Radical Prostatectomy, edn.. 2014. pp. 57–74.

[9] Mottet N, van den Bergh RCN, Briers E, Bourke L, Cornford P, De Santis M, Gillessen S, Govorov A, Grummet J, Henry AM, et al. EAU - ESTRO - ESUR - SIOG Guidelines on Prostate Cancer 2018. In: European Association of Urology Guidelines 2018 Edition. Volume presented at the EAU Annual Congress Copenhagen 2018, edn. Arnhem, The Netherlands: European Association of Urology Guidelines Office; 2018.

[10] Luiting HB, van Leeuwen PJ, Busstra MB, Brabander T, van der Poel HG, Donswijk ML, Vis AN, Emmett L, Stricker PD, Roobol MJ (2020). Use of gallium-68 prostate-specific membrane antigen positron-emission tomography for detecting lymph node metastases in primary and recurrent prostate cancer and location of recurrence after radical prostatectomy: an overview of the current literature. BJU Int.

[11] Bouchelouche K, Choyke PL, Capala J (2010). Prostate specific membrane antigen—a target for imaging and therapy with radionuclides. Discov Med.

[12] Zhang Q, Zang S, Zhang C, Fu Y, Lv X, Zhang Q, Deng Y, Zhang C, Luo R, Zhao X (2017). Comparison of (68)Ga-PSMA-11 PET-CT with mpMRI for preoperative lymph node staging in patients with intermediate to high-risk prostate cancer. J Transl Med.

[13] Yaxley JW, Raveenthiran S, Nouhaud FX, Samartunga H, Yaxley AJ, Coughlin G, Delahunt B, Egevad L, McEwan L, Wong D (2019). Outcomes of primary lymph node staging of intermediate and high risk prostate cancer with (68)Ga-PSMA positron emission tomography/computerized tomography compared to histological correlation of pelvic lymph node pathology. J Urol.

[14] Afshar-Oromieh A, Holland-Letz T, Giesel FL, Kratochwil C, Mier W, Haufe S, Debus N, Eder M, Eisenhut M, Schafer M (2017). Diagnostic performance of (68)Ga-PSMA-11 (HBED-CC) PET/CT in patients with recurrent prostate cancer: evaluation in 1007 patients. Eur J Nucl Med Mol Imaging.

[15] Afshar-Oromieh A, Haberkorn U, Eder M, Eisenhut M, Zechmann CM (2012). [68Ga]Gallium-labelled PSMA ligand as superior PET tracer for the diagnosis of prostate cancer: comparison with 18F-FECH. Eur J Nuclear Med Mol Imaging.

[16] Higgins JP, Thompson SG, Deeks JJ, Altman DG (2003). Measuring inconsistency in meta-analyses. BMJ.

[17] Reitsma JB, Glas AS, Rutjes AW, Scholten RJ, Bossuyt PM, Zwinderman AH (2005). Bivariate analysis of sensitivity and specificity produces informative summary measures in diagnostic reviews. J Clin Epidemiol.

[18] Hamza TH, Arends LR, van Houwelingen HC, Stijnen T (2009). Multivariate random effects meta-analysis of diagnostic tests with multiple thresholds. BMC Med Res Methodol.

[19] Song F, Khan KS, Dinnes J, Sutton AJ (2002). Asymmetric funnel plots and publication bias in meta-analyses of diagnostic accuracy. Int J Epidemiol.

[20] Herlemann A, Wenter V, Kretschmer A, Thierfelder KM, Bartenstein P, Faber C, Gildehaus FJ, Stief CG, Gratzke C, Fendler WP (2016). (68)Ga-PSMA positron emission tomography/computed tomography provides accurate staging of lymph node regions prior to lymph node dissection in patients with prostate cancer. Eur Urol.

[21] Obek C, Doganca T, Demirci E, Ocak M, Kural AR, Yildirim A, Yucetas U, Demirdag C, Erdogan SM, Kabasakal L (2017). The accuracy of (68)Ga-PSMA PET/CT in primary lymph node staging in high-risk prostate cancer. Eur J Nucl Med Mol Imaging.

[22] Gupta M, Choudhury PS, Hazarika D, Rawal S (2017). A comparative study of (68)Gallium-prostate specific membrane antigen positron emission tomography-computed tomography and magnetic resonance imaging for lymph node staging in high risk prostate cancer patients: an initial experience. World J Nucl Med.

[23] Yilmaz B, Turkay R, Colakoglu Y, Baytekin HF, Ergul N, Sahin S, Tugcu V, Inci E, Tasci AI, Cermik TF (2019). Comparison of preoperative locoregional Ga-68 PSMA-11 PET-CT and mp-MRI results with postoperative histopathology of prostate cancer. Prostate.

[24] Maurer T, Gschwend JE, Rauscher I, Souvatzoglou M, Haller B, Weirich G, Wester HJ, Heck M, Kubler H, Beer AJ (2016). Diagnostic efficacy of (68)Gallium-PSMA positron emission tomography compared to conventional imaging for lymph node staging of 130 consecutive patients with intermediate to high risk prostate cancer. J Urol.

[25] Budaus L, Leyh-Bannurah SR, Salomon G, Michl U, Heinzer H, Huland H, Graefen M, Steuber T, Rosenbaum C (2016). Initial Experience of (68)Ga-PSMA PET/CT imaging in high-risk prostate cancer patients prior to radical prostatectomy. Eur Urol.

[26] van Leeuwen PJ, Emmett L, Ho B, Delprado W, Ting F, Nguyen Q, Stricker PD (2017). Prospective evaluation of 68Gallium-prostate-specific membrane antigen positron emission tomography/computed tomography for preoperative lymph node staging in prostate cancer. BJU Int.

[27] Abdollah F, Karnes RJ, Suardi N, Cozzarini C, Gandaglia G, Fossati N, Bianchi M, Boorjian SA, Sun M, Karakiewicz PI (2014). Predicting survival of patients with node-positive prostate cancer following multimodal treatment. Eur Urol.

[28] Abdollah F, Sun M, Thuret R, Budäus L, Jeldres C, Graefen M, Briganti A, Perrotte P, Rigatti P, Montorsi F (2010). Decreasing rate and extent of lymph node staging in patients undergoing radical prostatectomy may undermine the rate of diagnosis of lymph node metastases in prostate cancer. Eur Urol.

[29] Galper SL, Chen MH, Catalona WJ, Roehl KA, Richie JP, D'Amico AV (2006). Evidence to support a continued stage migration and decrease in prostate cancer specific mortality. J Urol.

[30] Kawakami J, Meng MV, Sadetsky N, Latini DM, Duchane J, Carroll PR (2006). Changing patterns of pelvic lymphadenectomy for prostate cancer: results from CaPSURE. J Urol.

[31] Mandel P, Tilki D, Chun FK, Pristupa E, Graefen M, Klutmann S, Budaus L, Steuber T (2020). Accuracy of (68)Ga-prostate-specific membrane antigen positron emission tomography for the detection of lymph node metastases before salvage lymphadenectomy. Eur Urol Focus.

[32] Grivas N, van den Bergh RCN, Brouwer OR, KleinJan GH, Ramirez-Backhaus M, Wilthagen EA, van der Poel HG (2020). Pelvic lymph node distribution and metastases of prostate and bladder cancer: a systematic literature review and template proposal. World J Urol.

[33] Luchini C, Fleischmann A, Boormans JL, Fassan M, Nottegar A, Lucato P, Stubbs B, Solmi M, Porcaro A, Veronese N (2017). Extranodal extension of lymph node metastasis influences recurrence in prostate cancer: a systematic review and meta-analysis. Sci Rep.

[34] Motterle G, Ahmed ME, Andrews JR, Karnes RJ (2019). The role of radical prostatectomy and lymph node dissection in clinically node positive patients. Front Oncol.

[35] Rosiello G, Bandini M, Briganti A (2019). Salvage pelvic lymph node dissection for lymph node recurrent prostate cancer. Curr Opin Urol.

[36] Hövels AM, Heesakkers RA, Adang EM, Jager GJ, Strum S, Hoogeveen YL, Severens JL, Barentsz JO (2008). The diagnostic accuracy of CT and MRI in the staging of pelvic lymph nodes in patients with prostate cancer: a meta-analysis. Clin Radiol.

[37] Moher D, Liberati A, Tetzlaff J, Altman DG (2009). Preferred reporting items for systematic reviews and meta-analyses: the PRISMA statement. PLoS Med.

